# Factors associated with malaria chemoprophylaxis compliance among French service members deployed in Central African Republic

**DOI:** 10.1186/s12936-016-1219-4

**Published:** 2016-03-17

**Authors:** Marie-Aude Créach, Guillaume Velut, Franck de Laval, Sébastien Briolant, Luc Aigle, Catherine Marimoutou, Xavier Deparis, Jean-Baptiste Meynard, Bruno Pradines, Fabrice Simon, Rémy Michel, Aurélie Mayet

**Affiliations:** French Armed Forces Centre for Epidemiology and Public Health (CESPA), GSBdD, Marseille Aubagne-111, Avenue de la Corse-P 40026, 13568 Marseille cedex 02, France; INSERM, UMR912 (SESSTIM), 13006 Marseille, France; Operation Sangaris, Bangui, Central African Republic; Inter-Army Health Service Directorate, Cayenne, French Guiana France; Parasitology Laboratory, Institut Pasteur of French Guiana, Cayenne, French Guiana France; Research Unit on Emerging Infectious and Tropical Diseases, Aix Marseille University, UM 63, CNRS 7278, IRD 198, Inserm 1095, Marseille, France; Parasitology and Entomology Unit, Department of Infectious Diseases, Armed Forces Biomedical Research Institute, Brétigny sur Orge, France; National Reference Centre for Malaria, Marseille, France; Department of Infectious and Tropical Diseases, Laveran Armed Forces Teaching Hospital, Marseille, France; Ecole du Val-de-Grâce, Paris, France

**Keywords:** Armed forces, Malaria, Chemoprophylaxis, Compliance, Health education, Peer-to-peer reinforcement

## Abstract

**Background:**

Malaria is a public health concern in the French armed forces, with 400–800 cases reported every year and three deaths in the past 2 years. However, lack of chemoprophylaxis (CP) compliance is often reported among service members. The aim of this study was to explore factors associated with CP compliance.

**Methods:**

A retrospective study (1296 service members) was carried out among troops deployed in Central African Republic. Determinants of CP were collected by self-questionnaire. Socio-demographic variables, behavioural characteristics, belief variables, operational determinants such as troops in contact (TIC) and number of nights worked per week and peer-to-peer reinforcement were studied. Relationships between covariates and compliance were explored using logistic regressions (outcome: compliance as a dummy variable).

**Results:**

Chemoprophylaxis compliance was associated with other individual preventive measures against mosquito bites (bed net use, OR (odds ratio) = 1.41 (95 % CI [1.08–1.84]), and insecticide on clothing, OR = 1.90 ([1.43–2.51]) and malaria-related behaviours (taking chemoprophylaxis at the same time every day, OR = 2.37 ([1.17–4.78]) and taking chemoprophylaxis with food, OR = 1.45 ([1.11–1.89])). High perceived risk of contracting malaria, OR = 1.59 ([1.02–2.50]), positive perception of CP effectiveness, OR = 1.62 ([1.09–2.40]) and the practice of peer-to-peer reinforcement, OR = 1.38 ([1.05–1.82]) were also associated with better compliance. No association was found with TIC and number of nights worked.

**Conclusions:**

This study, which shows a positive relationship between peer-to-peer reinforcement and CP compliance, also suggests the existence of two main personality profiles among service members: those who seek risks and those who are health-conscious. Health education should be expanded beyond knowledge, know-how and motivational factors by using a comprehensive approach based on identification of health determinants, development of psychosocial skills and peer-to-peer reinforcement.

## Background

Every year, 25–30 million people travel to countries where malaria is present, and about 30,000 develop clinical malaria [1]. For non–immune travellers, the riskiest area is Sub-Saharan Africa.

At least 35,000 French service members are deployed or travel through malaria-endemic areas each year [2]. Malaria control strategies in the French armed forces are based on three combined strategies [3]: (1) *Anopheles* vector control using personal protection adapted to the troops’ field living conditions; (2) Chemoprophylaxis (CP) based on doxycycline monohydrate, which proved effective and well tolerated [4, 5]; (3) Management of cases through early diagnosis and appropriate treatment. These measures are associated with permanent health education of military personnel, epidemiological surveillance of malaria cases and monitoring of *Plasmodium* chemosusceptibility [3].

Despite these control measures, 400–800 malaria cases are recorded every year by the French Armed Forces Centre for Epidemiology and Public Health (CESPA) [6]. Three deaths were even reported in the last 2 years. Among the outbreaks that occurred overseas, the largest took place in Côte d’Ivoire in 2003, accounting for over 600 cases and an incidence of 97 cases per 1000 person-years [7–9].

During the first mandate (4 months) of Operation Sangaris, which started in Central African Republic in December 2013, 100 malaria cases were reported (equalized incidence: 150 p.1000 person-years). According to a case control study conducted following these events, the only factor significantly associated with malaria occurrence was lack of CP compliance (odds ratio (OR) = 6.5), no relationship being observed for other measures (insect repellent use, wearing of long sleeves and long trousers at night, use of a bed net and insecticide on clothing) (CESPA, unpublished data). This outbreak continued until November 2014, accounting for more than 240 cases, among which 70 were evacuated by helicopter to a field hospital and four were repatriated to France.

Previous research suggested that among French service members deployed in Africa, operational settings are associated with better CP compliance when compared to training settings [10]. Nevertheless, during Operation Sangaris, several epidemic peaks occurred concurrently to the most intense combat periods, as had already been described in Ivory Coast [9]. In a combat setting, service members move more frequently, work more often at night and are subjected to greater stress, which may lead to a lack of CP compliance.

Following these observations and in order to improve the French armed forces malaria control strategy, the present study aimed at exploring the factors associated with CP compliance among French service members on short-term deployment (i.e., 4 months) in Central African Republic, focusing on operational living conditions and reinforcing behaviours.

## Methods

### Study design and inclusion criteria

An observational retrospective study was carried out between October 11 and November 08, 2014, among the total workforce of the third military mandate of Operation Sangaris. A total of 2130 service members participated in this mission. The end of the mission included a 3-day, post-deployment well-being period in Dakar (Senegal) just before their return to France, aimed at helping them manage the transition from high-stress war zone to a peaceful home environment. This post-deployment period was applied to 13 successive groups. Among these groups, seven were randomly selected, accounting for 1296 service members.

All members of these groups were invited to participate in the study. A self-administered questionnaire was filled out by each individual. People who left the mission before the post-deployment period (medical or disciplinary repatriation during the mission) were not included in the study.

### Ethical issues

The protocol was approved by the South Mediterranean I Committee for the Protection of Persons (Notice no. RO—2014/10, 01/10/2014). The informed consent of each participant was obtained at the beginning of the study after a thorough explanation of its purpose. Subjects were free to refuse to participate.

### Data collection

The outcome was the reported compliance with malaria CP during the mission. This variable was initially coded in four classes: “no missed doses” during the mission as a whole, “less than one missed dose per month”, “between one and four missed doses per month” or “more than four missed doses per month”. Due to the number of covariates studied and to maximize the size of subgroups, the outcome was dichotomized: “correct” compliance was defined as “no missed doses” of daily drug intake during the mission as a whole (reference: at least one dose missed).

Several covariates that could be considered as potential determinants of compliance were recorded:

- Demographic variables included age (≤25 years vs >25), gender (female vs male), marital status (living with a partner vs not), having a child(ren) (yes vs no), healthcare personnel (yes vs no), rank (officer and noncommissioned officer (NCO) vs rank and file), management responsibilities (>10 subordinates vs ≤10 subordinates), length of service in the armed forces (≥6 years vs <6), number of previous mission(s) (≥4 and more vs <4), previous travel to other malaria-endemic areas (yes vs no) and previous malaria episode history (yes vs no).
- Mission characteristics included the tactical areas visited during the mission (“Acier”, “Magenta”, “de Boissieu” corresponding to combat troops; “DETLOG” corresponding to logistical support, “PCIAT” corresponding to armed forces staff and “others”, which combined French Army light aircraft crews and communication services: yes *vs* no for each area, a same subject being able to visit several during their mission), the number of “troops in contact” (TIC) (≥4 combat situations encountered vs <4), the average number of nights worked (≥2 nights per week vs <2) and bedtime during the mission (after midnight vs before or at midnight).
- Behavioural characteristics included tobacco (smoker vs no-smoker) and alcohol (≥4 units for one occasion vs <4) use and malaria-related behaviours: frequency of CP intake (at the same time *vs* at different times of the day), taking CP with food, wearing long sleeves and long trousers at night, use of a bed net, insect repellent and insecticide on clothing (“always” vs “not always” for each variable). The last behavioural characteristic was peer-to-peer reinforcement. This variable was built by integrating two variables: the number of occasions where the subject reminded other service members to take their CP (active reinforcement) and where the subject was reminded by other service members to take their CP (passive reinforcement). For both questions, answers could be “almost daily”, “more than three times per week”, “less than three times per week” and “never”. Efficient peer-to-peer reinforcement was defined as “almost daily” for active and passive reinforcements.
- Belief variables concerned several perceptions including risk of contracting malaria during their stay (high or very high vs none or low), severity of malaria (high or very high vs none or low), effectiveness of CP (sufficient or very effective vs ineffective or not effective enough) and individual attractiveness to mosquitoes (equal to other service members vs less and equal vs more).
- Health disorders included CP adverse events reported (yes vs no) and symptoms experienced during the mission (service members’ overall feeling and not symptoms directly linked with adverse events): sleep disorders, dizziness, vision problems, feeling of tiredness, digestive disorders, skin problems, irritation, stress, depressive mood, headaches and memory loss (≥3 days per week vs <3).
- Lastly, experience of public health measures included posters showing a prevention message (almost daily vs less), sufficient CP supply (yes vs no), information about CP use and adverse events (before or during the mission vs never).

### Statistical analyses

Data were recorded using Sphinx and were checked for consistency before statistical analysis using SAS version 9. Missing values affected less than 5 % of each variable, except for alcohol consumption (12 % missing values) and were coded as the analysis reference group. Integrating missing values in the other group was also tested for all variables to evaluate model stability.

Relationships between CP compliance and covariates were explored using regression logistic models. Explanatory variables with a *p* < 0.20 for at least one category of the outcome were screened for in the univariate analyses to identify eligible variables for the multivariate model. A backward stepwise procedure was used to identify the best-fit model by removing variables one at a time on the basis of a *p* value of >0.05 for at least one of the variable’s categories. Two variables that were particularly able to reflect operational constraint (number of TIC and average number of nights worked) were also maintained in the model.

## Results

### Population

**Fig. 1:**
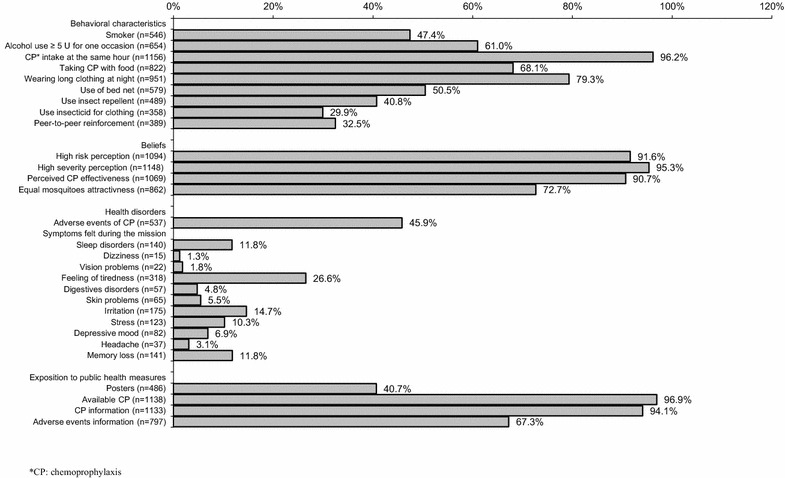
Distribution of behaviours, belief characteristics; symptoms experienced during the mission and public health measures (N = 1208). *CP chemoprophylaxis

Among the 1296 service members included, 1234 completed the questionnaire (95.2 % response rate). Twenty-six subjects were excluded, due to poorly filled-out questionnaires (N = 2) or missing data for the dependent variable (N = 24). The final sample thus totaled 1208 subjects. Fifty-two subjects reported malaria episodes. Average age was 30.5 years, standard deviation (SD) 7.7 years, extremes [19–57]. Sex ratio was 2.5 women/100 men. Rank and file represented 57.9 % (693/1197), non-commissioned officers 30.9 % (370/1197) and officers 11.2 % (134/1197). Age, gender and rank distributions were not significantly different between the sample and the population deployed in the field (*p* = 0.39, *p* = 0.17, *p* = 0.30). TIC average was 1.7, SD 2.34, extremes [0–30]. Average number of nights worked was 2.7 nights per week, SD 1.4, extremes [0–4]. Behaviours, beliefs and public health measures are presented in Fig. 1.

### Chemoprophylaxis compliance

Among the 1208 service members, 684 reported correct compliance (56.6 %). The non-compliant subjects included: “less than one missed dose per month” [reported by 374 service members (31.0 %)], “between one and four missed doses per month” [reported by 120 (9.9 %)] and “more than four missed doses per month” [reported by 30 (2.5 %)].

In univariate logistic models (Table 1), correct compliance was significantly associated with the following socio-demographic characteristics: age over 25 (odds ratio (OR) = 1.70 [1.33–2.17]); having a child(ren) (OR = 1.34 [1.06–1.70]); rank (OR = 2.68 [1.78–4.02] for officers and OR = 1.86 [1.44–2.41] for NCOs compared with rank and file); length of service over 6 years (OR = 1.71 [1.35–2.16]) and experience of more than four previous missions (OR = 1.36 [1.08–1.71]).

**Table 1:** Factors associated with malaria chemoprophylaxis use (no missed dose *vs* at least one missed dose during the mission). Logistic regression model (N = 1208 subjects)

				Univariate analyses			Multivariate analyses		
		Nb	%	OR	[95 % IC ]	*p*	OR	[95 % IC]	*p*
Sociodemographic characteristics									
Age (years) (ref: 18–25)									
26 and more		374	31.1	1.70	[1.33–2.17]	<10^−3^			
Gender (ref: male)									
Female		30	2.5	1.33	[0.63–2.83]	0.45			
Marital status (ref: no)									
Living with a partner		706	59.6	1.24	[0.98–1.57]	0.07			
Having child(ren) (ref: no)									
Yes		486	41.0	1.34	[1.06–1.70]	0.01			
Healthcare personal (ref: no)									
Yes		115	9.6	1.33	[0.89–1.97]	0.16			
Rank (ref: rank and file)									
NCOs		370	30.9	1.86	[1.44–2.41]		1.51	[1.12–2.03]	
Officer		134	11.2	2.68	[1.78–4.02]	<10^−3^	2.02	[1.27–3.31]	0.002
Management responsibilities (ref: no)									
Yes		122	10.2	1.36	[0.92–2.00]	0.12			
Length of service (year) (ref: 0–5)									
6 and more		704	58.9	1.71	[1.35–2.16]	<10^−3^			
Number of previous mission(s) (ref: 0–3)									
4 and more		543	45.3	1.36	[1.08–1.71]	0.01			
Previous travel malaria-endemic area (ref: no)									
Yes		927	77.2	1.21	[0.93–1.59]	0.16			
Previous episode malaria history (ref: no)									
Yes		74	6.2	0.71	[0.44–1.13]	0.15			
Mission characteristics									
Tactical areas visited (ref: not visited)									
Magenta		342	24.5	0.85	[0.66–1.09]	0.19			
Acier		208	14.9	1.12	[0.82–1.51]	0.48			
De Boissieu		309	22.1	1.21	[0.93–1.57]	0.16			
PCIAT		94	6.7	1.33	[0.86–2.05]	0.20			
DETLOG		189	13.5	0.55	[0.40–0.75]	0.0002	0.51	[0.36–0.74]	0.003
Other^a^		255	18.3	2.27	[1.68–3.06]	<10^−3^	1.60	[1.15–2.23]	0.005
Troops in contact (ref: 0–3)									
4 and more		310	2.8	0.81	[0.62–1.05]	0.11	0.81	[0.60–1.09]	0.16
Average number of nights worked (ref: 0–1 night/week)									
2 nights and more/week		882	73.9	0.60	[0.46–0.79]	0.0002	0.91	[0.66–1.26]	0.57
Bedtime during the mission (ref: before or at midnight)									
After midnight		59	5.2	0.46	[0.27–0.78]	0.004			
Behavioural characteristics									
Tobacco consumption (ref: non smoker)									
Smoker		546	47.4	0.60	[0.48–0.76]	<10^−3^	0.77	[0.60–0.99]	0.04
Alcohol use (ref: 0–4 unit(s) for one occasion)									
5 units and more for one occasion		654	61.0	0.74	[0.58–0.95]	0.02			
Rhythm of CP intake (ref: different times)									
At the same time every day		1156	96.2	3.47	[1.81–6.67]	<10^−3^	2.37	[1.17–4.78]	0.02
Taking CP with food (ref: not always)									
Always		822	68.1	1.85	[1.45–2.37]	<10^−3^	1.45	[1.11–1.89]	0.01
Wearing long clothing at night (ref: not always)									
Always		951	79.3	1.30	[0.98–1.72]	0.06			
Use of bed net (ref: not always)									
Always		579	50.5	1.66	[1.31–2.10]	<10^−3^	1.41	[1.08–1.84]	0.01
Insect repellent (ref: not always)									
Always		489	40.8	1.30	[1.03–1.64]	0.03			
Insecticide for clothing (ref: not always)									
Always		358	29.9	1.98	[1.53–2.57]	<10^−3^	1.90	[1.43–2.51]	<10^−3^
Peer-to-peer reinforcement (ref: less)									
Almost daily		389	32.5	1.37	[1.07–1.76]	0.01	1.38	[1.05–1.82]	0.02
Beliefs									
Risk to contract malaria (ref: none or low)									
High or very high		1094	91.6	1.65	[1.09–2.49]	0.02	1.59	[1.02–2.50]	0.04
Severity of malaria (ref: ineffective or not enough)									
Serious or very serious		1148	95.3	1.53	[0.89–2.62]	0.12			
Effectiveness of CP (ref: ineffective or not enough)									
Enough or very effective		1069	90.7	1.88	[1.26–2.79]	0.002	1.62	[1.09–2.40]	0.02
Attractiveness for mosquitoes (ref: equal)									
Less		178	15.0	0.80	[0.58–1.10]				
More		146	12.3	0.81	[0.57–1.15]	0.2446			
Health disorders									
Adverse events (AE) of CP (ref: no)									
Yes		537	45.9	0.70	[0.55–0.88]	0.002			
Symptoms felt/no linked with AE (ref: 0–2 days/week)									
Sleep disorders									
3 days and more/week		140	11.8	0.93	[0.65–1.33]	0.70			
Dizziness									
3 days and more/week		15	1.3	1.14	[0.40–3.23]	0.80			
Vision problems									
3 days and more/week		22	1.8	2.66	[0.97–7.26]	0.05	3.84	[1.25–11.83]	0.02
Feeling of tiredness									
3 days and more/week		318	26.6	0.58	[0.45–0.75]	<10^−3^	0.67	[0.50–0.90]	0.01
Digestives disorders									
3 days and more/week		57	4.8	0.54	[0.31–0.92]	0.02			
Skin problems									
3 days and more/week		65	5.5	0.59	[0.36–0.98]	0.04			
Irritation									
3 days and more/week		175	14.7	0.49	[0.35–0.68]	<10^−3^			
Stress									
3 days and more/week		123	10.3	0.61	[0.42–0.88]	0.01			
Depressive mood									
3 days and more/week		82	6.9	0.60	[0.38–0.94]	0.03			
Headache									
3 days and more/week		37	3.1	0.57	[0.29–1.11]	0.09			
Memory loss									
3 days and more/week		141	11.8	0.51	[0.35–0.72]	0.0001	0.67	[0.45–0.99]	0.05
Exposition to public health measures									
Posters with prevention messages (ref: less)									
Almost daily		486	40.7	1.28	[1.01–1.61]	0.04			
CP sufficient availability (ref: no)									
Yes		1138	96.9	2.39	[1.20–4.77]	0.01			
Information about CP (ref: never)									
Before or during the mission		1133	94.1	1.65	[1.02–2.67]	0.04			
Information about the AE of CP (ref: never)									
Before or during the mission		797	67.3	1.34	[1.05–1.72]	0.02			

*NCOs* non-commissioned officers, *CP* chemoprophylaxis

^a^Other: combining French Army’s light aircraft crews and communication services

With regard to mission characteristics, membership in DETLOG (OR = 0.55 [0.40–0.75]) and in other units (OR = 2.27 [1.68–3.06]) was associated with better compliance, while subjects who reported more than two nights worked per week (OR = 0.60 [0.46–0.79]) and a bedtime after midnight (OR = 0.46 [0.27–0.78]) tended to be less compliant.

Psychoactive substance use was associated with a lack of compliance (OR = 0.60 [0.48–0.76] for tobacco and OR = 0.74 [0.58–0.95] for alcohol). However, some malaria-related behaviours were positively associated with compliance: taking CP at the same time every day (OR = 3.47 [1.8–6.67]), always taking CP with food (OR = 1.85 [1.45–2.37]), bed net use (OR = 1.66 [1.31–2.10]); insect repellent use (OR = 1.30 [1.03–1.64]); insecticide use on clothing (OR = 1.98 [1.53–2.57]), and peer-to-peer reinforcement (OR = 1.37 [1.07–1.76]). Improved compliance was also observed among subjects who perceived a significant individual malaria risk (OR = 1.65 [1.09–2.49]) and that CP was effective (OR = 1.88 [1.26–2.79]).

Subjects who reported CP adverse events (OR = 0.70 [0.55–0.88]) and experienced several symptoms during their mission (tiredness (OR = 0.58 [0.45–0.75]), digestives disorders (OR = 0.54 [0.31–0.92]), skin problems (OR = 0.59 [0.36–0.98]), irritation (OR = 0.49 [0.35–0.68]), stress (OR = 0.61 [0.42–0.88]); depressive mood (OR = 0.60 [0.38–0.94]) and memory loss (OR = 0.51 [0.35–0.72])) were less likely to be compliant.

Lastly, some public health measures were associated with better compliance: posters with prevention messages (OR = 1.28 [1.01–1.61]), CP availability (OR = 2.39 [1.20–4.77]), information about CP use (OR = 1.65 [1.02–2.67]) and adverse events (OR = 1.34 [1.05–1.72]).

The factors that remained significant in multivariate analysis were rank, membership in DETLOG (OR = 0.51 [0.36–0.74], *p* = 0.003) and in others tactical areas (OR = 1.60 [1.15–2.23], *p* = 0.005), tobacco use (OR = 0.77 [0.60–0.99], *p* = 0.04), malaria-related behaviours (taking CP at the same time: OR = 2.37 [1.17–4.78], *p* = 0.02; always taking CP with food: OR = 1.45 [1.11–1.89], *p* = 0.01; bed net use: OR = 1.41 [1.08–1.84], *p* = 0.01; insecticide on clothing: OR = 1.90 [1.43–2.51], *p* = <10^−3^); peer-to-peer reinforcement (OR = 1.38 [1.05–1.82], *p* = 0.02); high perceived risk of contracting malaria during the stay (OR = 1.59 [1.02–2.50], *p* = 0.04); CP effectiveness (OR = 1.62 [1.09–2.40], *p* = 0.02); and three symptoms (vision problems: OR = 3.84 [1.25–11.83], *p* = 0.02; tiredness: OR = 0.67 [0.50–0.90], *p* = 0.01; and memory loss: OR = 0.67 [0.45–0.99], *p* = 0.05). TIC and number of nights worked were not significantly associated with compliance. The final model likelihood did not significantly differ from those of the stepwise model without TIC and number of nights worked. The results of both models were very close in terms of determinants and association strength.

## Discussion

The main finding of this study is that CP compliance appears strongly related to compliance with other prophylactic measures against malaria, suggesting a common receptivity to health preservation that may be observed among some individuals. This behaviour also appears to be associated with correct perception of malaria risk. On the other hand, subjects who use psychoactive substances showed a lack of CP compliance, suggesting an “opposite” personality that may reflect generalized risk-taking behaviours. This risk-taking personality is in line with literature about polysubstance use among the military and civilians [11–13]. Moreover, recent research has also suggested relationships between addictive behaviours and poor vaccine acceptance in the French general population [14]. Another key finding is that peer-to-peer reinforcement constitutes a determinant of better CP compliance, a result not previously published in the area of malaria prevention.

Previous studies about the relationships between CP compliance and other preventive measures against mosquito bites are contradictory. Whereas some studies showed no association [15] or even competition [16] with the use of personal protection measures, two other studies [17, 18] reported that compliance with CP was positively associated with anti-mosquito measures, in line with the present study. In addition to the traditional malaria-related behaviours, the present study also suggests that more compliant subjects are also more likely to believe in CP effectiveness and to follow recommendations for doxycycline-monohydrate pill taking: pills should be taken at the same time each day, concurrently with food. This indicates that compliance with a prevention measure is multifactorial, involving three factors: knowledge, know-how and motivation. These factors are those that have been identified as targets by health education programs in the French armed forces [19].

No difference in CP compliance was found according to perceived severity of malaria, which is not in line with another study among French service members deployed in Africa that showed that perceived high severity of malaria was associated with correct compliance [17]. Yet, the risk of contracting malaria was perceived as high during the mission and was associated with correct compliance. This result was expected and other military studies reported that perceived threat due to mosquitoes or high perceived risk of malaria were associated with CP compliance [18, 20, 21]. In civilian studies, it has also been shown that travellers who perceived themselves as being at high risk of developing malaria after returning home [22] or travellers in high-risk malaria-endemic areas who had correctly perceived the level of risk [15] were more often compliant with CP. These results suggest that prevention messages about the perception of severity will not be directly effective to incite people to change their behaviour [23]. In contrast, malaria risk perception and effectiveness of CP will be effective drivers for prevention actions.

Younger age, smoking, high-risk alcohol consumption and bedtime after midnight (the two last variables being only significant in univariate analysis) were associated with low CP compliance. These criteria match certain personality traits and dimensions, such as novelty-seeking, impulsivity, independent behaviour, risk-taking, anti-conformism, lack of persistence and extroversion [24], which appear to be linked to both genetic factors and environment [25]. It could be hypothesized that harsh field conditions, which have already been described as stress inducers [26], may exacerbate the environmental impact. Some previous studies about alcohol use are in line with this shared influence of individual and environmental factors, suggesting that service members may not only use alcohol as an individual strategy to cope with underlying depressive disorders, but also as a collective strategy to increase group bonding to cope with the difficulties of military profession [27–29]. This generalized risk-taking behaviour, likely to be observed among service members, appears to counterbalance the above-mentioned common receptivity to health preservation. The fact that peer-to-peer reinforcement was associated with better CP compliance suggests new approaches that could be implemented for health education: The receptive subjects could be trained to deliver prevention messages to the less motivated ones in order to improve this reinforcement.

Prevention messages given with posters and collective information about CP were associated with CP compliance, but did not remain significant in multivariate analysis. This suggests that underlying personality could be a determinant of CP compliance regardless of the current health education received. Other military studies have highlighted the importance of malaria counseling and personalized briefings [18, 30], but these methods, which target motivation and knowledge, need to be improved by using a comprehensive approach based on health determinants and psychosocial skills. In this way, peer-to-peer reinforcement should be encouraged.

The present study did not show any association between previous medical history of malaria and CP compliance. A previous study [17] showed that a previous medical history of malaria was associated with a present lack of compliance. This suggests that having experienced an episode of clinical malaria was not in itself contributing to the adoption of prophylactic behaviours [22]. The belief that a previous medical history of malaria is equivalent to an immunization has to be investigated.

Lastly, the present study did not find any association between CP compliance and TIC or average nights worked per week, unlike previous results suggesting an effect of initial combat intensity [31]. It may indicate that these variables do not sufficiently reflect variations over time in operational conditions. However, tiredness is associated with decreased compliance: it could be an indirect marker of the effect of operations. A further study should include tools already evaluated in order to explore stress in the military population [32].

Several limitations have to be addressed. Compliance was measured based on self-administered questionnaire, which may have overestimated the rate of correct compliance, as previously shown for anti-viral therapy [33] or malaria CP [34]. However, the accuracy of self-questionnaires for exploring CP compliance was assessed in previous research in the French armed forces (kappa coefficient = 0.65 between self-report and CP plasma concentration) [35]. While rank distribution was close to those of the French armed forces as a whole, young males were more represented in the field than in the French armed forces in general [36]. Nevertheless, the sample appeared representative of the French service members deployed in Central African Republic.

The prevalence of correct CP compliance in this study was slightly higher than the average compliance for the same type of population in inter-tropical Africa [17], and was close to that of other military [20] or civilian studies [15, 16]. Several determinants of compliance were similar to those identified among other armies or civilian travellers as well. Even if certain results cannot be directly extrapolated to civilians, they are useful for identifying key factors to enhance compliance with malaria CP.

## Conclusions

Although individual and collective public health measures have already been implemented against malaria, the French Military Health Service must expand health education programmes beyond knowledge, know-how and motivational factors by using a comprehensive approach based on identification of health determinants and development of psychosocial skills. The present study highlights two main personality profiles among service members: those who seek risks and those who are health-conscious. These profiles are in line with those previously described by Russell [37]: subjects drawn to the security of the military system and subjects drawn by the potential for excitement or adventure. Peer-to-peer reinforcement is a promising group approach to improving chemoprophylaxis compliance.
